# Descending necrotizing mediastinitis associated with *Lactobacillus plantarum*

**DOI:** 10.1186/1471-2334-13-398

**Published:** 2013-08-29

**Authors:** Takahito Nei, Shunta Inai, Iwao Mikami, Akira Sato, Junichi Okamoto, Kazuhiko Yokoshima, Munenaga Nakamizo, Shuji Haraguchi, Kazunari Sonobe, Ryoichi Saito

**Affiliations:** 1 Department of Infection Control and Prevention, Nippon Medical School, 1-1-5 Sendagi, Bunkyo-ku, Tokyo 113-8603, Japan; 2 Department of Otorhinolaryngology, Head and Neck Surgery, Nippon Medical School, 1-1-5 Sendagi, Bunkyo-ku, Tokyo 113-8603, Japan; 3 Department of Surgery (Division of Thoracic Surgery), Nippon Medical School, 1-1-5 Sendagi, Bunkyo-ku, Tokyo 113-8603, Japan; 4 Department of Clinical Laboratory, Nippon Medical School, 1-1-5 Sendagi, Bunkyo-ku, Tokyo 113-8603, Japan; 5 Department of Microbiology and Immunology, Tokyo Medical and Dental University, 1-5-45, Yushima, Bukyo-ku, Tokyo 113-8510, Japan; 6 Department of Internal Medicine (Division of Respiratory, Medicine, Infection and Oncology), 1-1-5 Sendagi, Bunkyo-ku, Tokyo 113-8603, Japan

**Keywords:** Descending necrotizing mediastinitis (DNM), Laryngeal cancer, Thoracoscopic surgery, *Lactobacilli*

## Abstract

**Background:**

Descending necrotizing mediastinitis (DNM), a severe infection with a high fatality rate, develops in mediastinal spaces due mainly to deep cervical abscesses. The majority of causative microbes of DNM are *Streptococci* and oral anaerobes. DNM associated with *Lactobacillus*-infection is rather rare.

**Case presentation:**

A 69-year-old male with an unremarkable past medical history was referred to our hospital for surgical resection of advanced laryngeal cancer. Full examination revealed a neck abscess and DNM with a background of untreated diabetes mellitus. Initially, he was treated with meropenem. However, *Lactobacillus plantarum* was isolated from surgical drainage of a mediastinal abscess. Despite using antibiotics capable of eradicating all isolates with susceptibilities not differing significantly from those of the neck and mediastinal abscesses, we attributed DNM to the *L. plantarum* detected only in the mediastinal abscess. After DNM treatment, he underwent total pharyngolaryngectomy with bilateral neck dissection followed by reconstruction using free jejunum. He was discharged fully recovered.

**Conclusion:**

We concluded that *L. plantarum* as the sole cause of the mediastinal abscess in the present case cannot be ruled out. As the number of immunocompromised patients increases, we should be cautious regarding this “familiar” microbe.

## Background

Infectious mediastinitis is a life-threatening though rare intrathoracic infection. One of the severe forms of this infection is descending necrotizing mediastinitis (DNM), which is characterized by diffuse necrosis that occurs as a complication of deep cervical infection or an esophageal disorder spreading along the deep fascial planes into the mediastinum [1,2]. The main etiological factors of DNM are oropharyngeal abscesses. However, fluid collections, abscesses, cellulitis and necrosis are among the local changes and lesions observed. DNM often has a fulminant course, rapidly progressing to sepsis and frequently death. Even in this era of appropriate surgical interventions and highly effective antibiotics, DNM still carries high mortality rates of 10~40% [2]. The majority of causative microbes of DNM are the same as those of oropharyngeal infections; i.e., *Streptococci* and oral anaerobes [2]. The majority of reported DNM cases had polymicrobial infections including both aerobes and anaerobes, reflecting the indigenous microbiological flora of oral or pharyngeal sites. DNM associated with *Lactobacillus*-infection was previously described [3], and *L. catendforme* and *L. jensenii* were reportedly isolated with *Streptococci* and oral anaerobes from mediastinal pus obtained surgically.

*Lactobacilli* are Gram-positive bacilli that are usually innocuous and exist in daily living environments as probiotics worldwide. They also constitute normal flora of the human vagina, oropharynx, and gastrointestinal tract [4]. However, *Lactobacilli* can cause infectious diseases, such as bacteremia [5] and endocarditis [6-8], as well as splenic [9,10] and hepatic abscesses [11], in humans. Moreover, *Lactobacilli* have emerged as pathogenic microbes in both immunocompetent and immunocompromised hosts [12,13].

*L. plantarum* has also been regarded as a probiotic, as have *L. caseii* and *L. rhamnosus*. They are both commonly isolated as harmless environmental microorganisms, and are used for food fermentation [13]. There are an especially large number of traditional fermented foods in Japan, aside from cheese and yogurt, i.e., Natto and Nuka-zuke (a type of pickle). However, *Lactobacilli* have recently been identified as potential emerging infectious microorganisms in immunocompromised patients, especially those with cancers and receiving chemotherapy or those with impaired glucose metabolism, including diabetes mellitus, and corticosteroid-treated patients.

## Case presentation

A 69-year-old male with no past history of major illness was referred to our hospital for surgical resection of advanced laryngeal cancer. He had a fever (over 38.5°C) and neck swelling at the first visit to our hospital. Emergency computed tomography (CT) of the neck showed a tumor-like lesion and an abscess in the anterior neck region. This lesion was near the thyroid gland. He underwent surgical incision and drainage, and levofloxacin (500 mg/day) was administered for 5 days after the first hospital visit. After incision and drainage, microbiological culture including anaerobic studies of the abscess showed *Fusobacterium necrophorum*, *Prevotella melanigenica*, and *Streptococcus anginosus/milleri* groups. We planned radical surgery for the laryngeal cancer, but prioritized treatment of the abscess.

**Figure 1:**
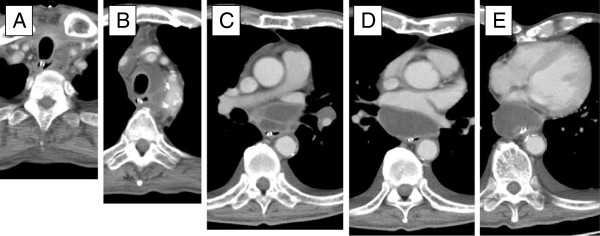
**Chest CT scan on admission.** From head to abdomen **(A to E)**, low-density masses in the mediastinal region are recognizable in all scans.

**Figure 2:**
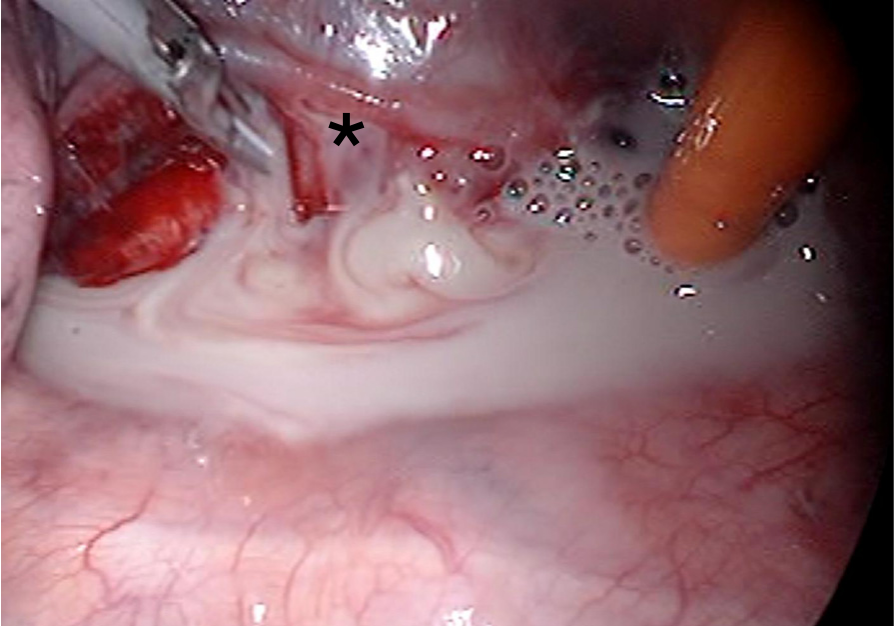
**Photograph taken during thoracoscopic surgical incision and drainage.** The apex of the right lung is on the left and the diaphragm is on the right. The asterisk indicates a mediastinal abscess located beneath the pulmonary vein.

**Figure 3:**
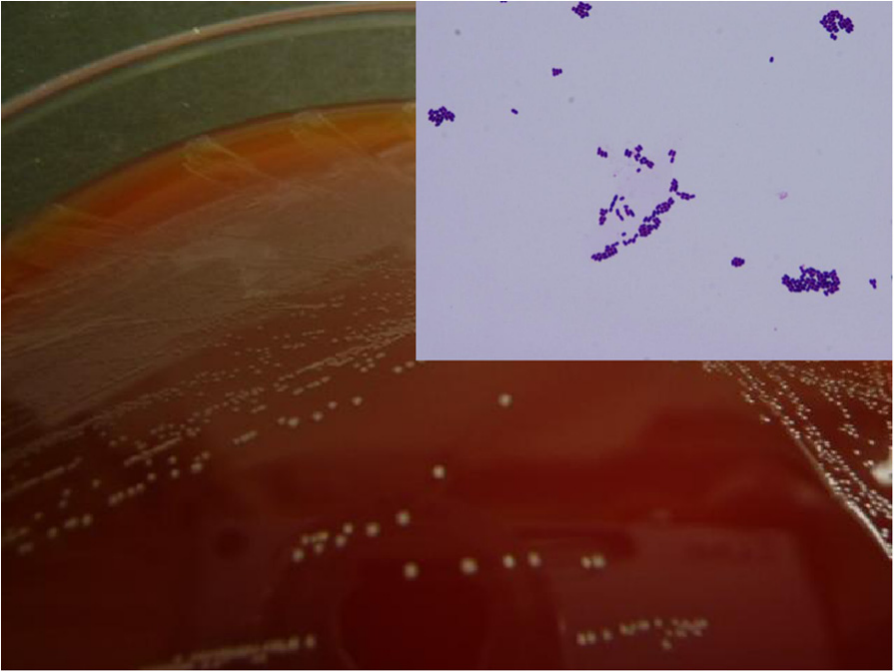
**Isolation of colonies from the mediastinal abscess following surgical incision, and microscopic view of isolates (the upper right panel, original magnification of × 1,000).** All colonies are less than 0.5 mm in diameter with α-hemolysis and appear yellow with slightly gray areas in blood agar medium. Isolates were found to be short Gram-positive bacilli by microscopic study.

On admission, his physical findings were normal except for a slight fever. His laboratory findings included a white blood cell (WBC) count of 19,500/μl (87.9% neutrophils) and C-reactive protein level of 24.63 mg/dl. Plasma glucose was 106 mg/dl and hemoglobin A1c was 8.1%. His medical history was otherwise unremarkable with no evidence of immunosuppression. After hospital admission, according to the susceptibilities of cultured microbes, we switched the antibiotic to meropenem (500 mg/q.12.h). However, pus from the neck abscess showed no change and his biochemical data did not improve, despite regular incisions and debridement of necrotic tissue. On the 4th hospital day, we again cultured necrosis tissue from the neck, but no microbes were isolated. Based on the diagnosis of diabetes and high suspicion of mediastinal abscess, he underwent thoracic examination on the 6th hospital day. Chest CT scan (Figure 1) showed a multi-segmented low density mass in the mediastinum. Based on the laboratory findings and radiological imaging, he was diagnosed as having descending necrotizing mediastinitis (DNM), and immediately underwent mediastinal drainage by thoracoscopic surgery with 3 ports in the right thoracic wall (Figure 2). Contents of the abscess were sampled by direct puncture of the abscess site employing a sterile anaerobic maneuver with disinfected devices. Smear studies of surgical samples showed the presence of Gram-positive bacilli in the background of WBCs without bacterial phagocytosis. Samples were cultured on 5% sheep blood agar (Eiken Chemical, Tokyo, Japan), chocolate agar (Beckton Dickinson, NJ), McConkey agar (Oriental Yeast, Tokyo, Japan), Brucella HK agar (Kyokuto Pharmaceutical Industrial, Tokyo, Japan), and HK semi-solid medium (Kyokuto Pharmaceutical Industries). We performed 48-hour aerobic cultures at 35°C using 5% sheep blood agar, chocolate agar, and McConkey agar. We also performed anaerobic cultures for 48 hours at 35°C using Brucella HK agar. Gram-positive bacilli grew on 5% sheep blood agar (Figure 3), chocolate agar, and McConkey agar but only under aerobic conditions. No other microbes grew in these media. The obtained isolates were negative for catalase, and neither spores nor branch formation was observed. Initially, we identified the isolates as *Lactobacillus casei* by BD BBL CRYSTAL™ (Becton Dickinson). Determination of MICs by microbroth dilution antimicrobial susceptibility testing (Walkaway™ Plus Systems, Siemens, Munich, Germany) was performed according to the sample preparation and reading conditions recommended by the supplier. In short, isolated colonies were suspended in 3 ml of Inoculum Water (Siemens) until a density corresponding to the McFarland standard 0.5 (≒1.5 × 10^8^ cfu/ml) was obtained. We then added 100 μl to Mueller-Hinton broth supplemented with 3% lysed horse blood (Siemens), and determined MICs using the MICroFast™ series, Strepto MF5 panel (Siemens). Susceptibilities (MIC μg/ml) to several antibiotics were: penicillin G 8, ampicillin 2, cephazolin ≤8, cefotiam 16, imipenem ≤1, meropenem 0.5, gentamicin ≤1, erythromycin ≤0.25, clindamycin ≤0.5, minocyline ≤2, levofloxacin 4, trimethoprim/sulfamethoxazole ≤1, vancomycin >16, and rifampicin ≤1. Though administration of meropenem was initiated upon diagnosis of DNM, treatment was changed to ampicillin (12 g/day) as de-escalation therapy based on susceptibility testing on the 18th hospital day. We washed the abscess daily via the drain until the 42nd hospital day, and on the 45th hospital day, antibiotic administration was completed. During chest drainage therapy, we cultured neither gram-positive bacilli nor any other microbes from multiple pleural effusion samples. However, it was unclear whether other infectious sites existed because no other samples, i.e. blood or oral mucosa, were cultured. On the 63rd hospital day, he underwent total pharyngolaryngectomy with bilateral neck dissection followed by reconstruction using free jejunum. On the 117th hospital day, he was discharged in good condition, having received neither adjuvant chemotherapy nor radiation.

Though the isolates were identified as *L. casei* employing conventional biological techniques, we accurately identified the strain with 16 s ribosomal RNA genotyping, as previously described [14], and a similarity search was conducted using the BLAST program (DDBJ, Shizuoka, Japan). The results (1,501 bp; GenBank accession no. AB755630) showed 100% similarity to the reference strain of *Lactobacillus pentosus* (GenBank accession no. AJ292254) and *Lactobacillus plantarum* (GenBank accession no. AL935263). We finally confirmed the identification of *L. plantarum* using the *recA* sequence [15]. The results (313 bp; GenBank accession no. AB755631) showed 100% identity with *L. plantarum* (GenBank accession no. AL935263) [similarity to *L. pentosus* (GenBank accession no. AJ292254)], ultimately confirming *L. plantarum* infection.

## Discussion

The origin of the abscess in our patient was difficult to identify, but we suspect the most likely source to have been his oropharyngeal flora. Mediastinal abscess formation is known to be closely related to cervical abscess development and in this case appeared to be comprised of polymicrobial infectious organisms resembling isolates from the neck abscess. It was reasonable to consider the infectious source in this case to most likely have been the oropharyngeal lumen in the view of both the neck abscess and DNM. If so, in this case, the mediastinal abscess was due not only to *L. plantarum* but also to *F. necrophorum*, *P. melanogenica*, and *Str. anginosus/milleri* groups, cultured from the neck abscess before the diagnosis of DNM. However, only *L. plantarum* was obtained from the mediastinal abscess. No *L. plantarum* was isolated from the neck abscess. It was unclear why only *L. plantarum* was isolated from the mediastinal abscess by direct puncture via thoracic surgery.

We suspected that the phenomenon observed in this patient was attributable to the pharmacodynamics of antibiotics. We initially treated the neck abscess with meropenem, which effectively eradicated all of these isolates. Though we did not measure the MICs of neck isolates, according to previous records, the susceptibilities of organisms from the neck cultures to meropenem were (MIC_90_, μg/ml): *F. necrophorum* 0.25 [16], *P. melanogenica* 0.25 [17], and *Str. anginosus /milleri* groups 0.12 [18]. The MIC_90_ and MIC of *L.plantarum* obtained from thoracic surgery (0.5) did not differ significantly. If there was no difference in antibiotic shift into tissue between the neck and mediastinal abscesses, *L. plantarum* would also be eradicated, along with the other organisms isolated, by meropenem. However, only *L. plantarum* was isolated from the surgical specimen, despite using the same culture method as that employed for the neck abscess. Though the majority of DNM cases are attributed to polymicrobial infection and we obtained other isolates which may have been involved in DNM from the neck site, we concluded that *L. plantarum* alone may have been responsible for the present mediastinal abscess.

On the other hand, lactate production and the resulting pH decreasing are powerful antimicrobial activities of *Lactobacillus* spp. against other microorganisms. Among these secies, *L. plantarum* can produce not only lactate but also hydrogen peroxide [19,20], providing protection from manganese catalase but not hem catalase [21]. We thus speculated that a high concentration of hydrogen peroxide produced by *L. plantarum* inhibited the growth of other microorganisms in mediastinal lymph nodes.

## Conclusion

We experienced a case of DNM case associated with an extraordinarily rare microbe, *L. plantarum.* The present case underscores the importance of being aware of the possibility of *Lactobacilli* as pathogenic microbes. Furthermore, as the number of immunocompromised patients increases, we should be cautious regarding this “familiar” microbe. (1642 words).

### Consent

Written informed consent was obtained from the patient for publication of this Case Report and any accompanying images. A copy of the written consent form is available for review by the Editor of this journal.

## Competing interests

The authors declare that they have no competing interests.

## Authors’ contributions

Conception and drafting of the report; TN, IM, KY, KS and RS, Thoracic surgical management; IM, AS, JO and SH, Pharyngolaryngeal surgical management; SI, KY and MN. Clinical treatment and antimicrobial therapy; TN, IM, AS and JO, Genomic identification of isolates; RS. All authors read and approved the final manuscript.

## Pre-publication history

The pre-publication history for this paper can be accessed here:

http://www.biomedcentral.com/1471-2334/13/398/prepub
